# Innate receptors modulating adaptive T cell responses: KIR-HLA interactions and T cell-mediated control of chronic viral infections

**DOI:** 10.1007/s00251-023-01293-w

**Published:** 2023-01-31

**Authors:** Laura Mora-Bitria, Becca Asquith

**Affiliations:** Department of Infectious Disease, Faculty of Medicine, Imperial College London, London, UK

**Keywords:** Chronic viral infections, KIR, HLA, AICD, CD8 + T cells, NK cells

## Abstract

Killer-cell immunoglobulin-like receptors (KIRs) are mainly expressed on natural killer (NK) cells and are key regulators of innate immune responses. NK cells are the first responders in the face of infection and help promote placentation during pregnancy; the importance of KIRs in these NK-mediated processes is well-established. However, mounting evidence suggests that KIRs also have a prominent and long-lasting effect on the adaptive immune system. Here, we review the evidence for the impact of KIRs on T cell responses with a focus on the clinical significance of this interaction.

**Supplementary Information:**

The online version contains supplementary material available at 10.1007/s00251-023-01293-w.

## Introduction

Host-parasite interactions represent a major evolutionary force; pathogens acquire new immune evasion strategies, and in turn, in response to this ever-changing challenge, the set of immune defences diversifies. The appearance of an adaptive immune system in jawed vertebrates is considered to be the pinnacle of this diversification process. Indeed, adaptive CD8 + T cells play a leading role against viral infections, one of the major sources of selective pressure in humans (Fumagalli et al. 2011). T cells express somatically rearranged T cell receptors (TCRs) that can recognize viral peptides presented by human leucocyte antigen (HLA) molecules. TCR recognition is not sufficient to initiate a T cell response. Additional innate signals are required to activate and orchestrate adaptive responses (Janeway and Medzhitov 2002). This interdependence between the two defense arms has blurred the classical dichotomous view between innate and adaptive systems, and beyond the initiation of the adaptive response, functional definitions of innate and adaptive immunity are being relaxed (Kvell et al. 2007; Terrazzano and Carbone 2013). Here, we review a family of innate germline-encoded immune receptors, the inhibitory killer-cell immunoglobulin-like receptors (iKIRs) and their role in modulating the adaptive, somatically varying T cell immune response.

We focus this review on two main areas: evidence for KIR modulation of T cell responses and the clinical significance of KIR modulation of T cell responses.

## The diverse KIR gene family

KIRs are a family of activating and inhibitory receptors predominantly expressed in natural killer (NK) cells but also in subsets of late-stage differentiated T cells. KIRs are encoded by a polygenic gene family located in chromosome *19q13.4* containing up to 13 activating and inhibitory KIR genes with a high degree of homology. Each KIR can be distinguished by the number of extracellular immunoglobulin-like domains: *KIR2D*- genes encode receptors with 2 immunoglobulin domains whereas *KIR3D*- genes encode for receptors with 3 domains. Activating KIRs (aKIRs) are characterized by a short cytoplasmatic tail with an ITAM motif (e.g. *KIR2DS2*, S for short). In contrast, iKIRs have a long cytoplasmatic tail containing ITIM motifs (e.g. *KIR2DL2*, L for long). An exception to this rule is *KIR2DL4* which displays features of both activating and inhibitory isoforms; despite having an ITIM motif in its long cytoplasmatic tail, *KIR2DL4* contains a transmembrane activation motif and is considered an aKIR (Long et al. 2013). The ligands for inhibitory KIR and some activating KIRs include HLA class I molecules (Pende et al. 2019).

Several layers of genetic variation shape functional KIR diversity. The first layer of diversity is KIR gene content. Individuals vary in the number of activating and inhibitory KIR genes they carry in their genomes. Consequently, KIR diversity is created by copy number variation due to the high rates of recombination; this highly homologous and gene-dense region results in dozens of structural variants, or haplotypes, found across populations (Traherne et al. 2010). KIR haplotypes have been broadly grouped according to their gene content into group A and group B haplotypes. Group A haplotypes contain several iKIR genes, namely *KIR2DL1*, *KIR2DL2/L3* and *KIR3DL1*, in addition to the framework genes *KIR3DL2*, *KIR3DL3* and *KIR2DL4*. Group B haplotypes show more structural diversity than group A haplotypes and contain more aKIRs (Pyo et al. 2010; Uhrberg et al. 1997). In addition to the gene content variation, KIR epigenetic regulation shapes KIR repertoires (Manser et al. 2015); both NK and T cells can express one or more KIRs in a stochastic fashion and independently of HLA class I genotype (Björkström et al. 2012; Valiante et al. 1997).

A second level of diversity is the vast amount of allelic variation displayed by KIR genes. The greatest number of alleles has been reported for *KIR3DL1* and *KIR3DL3*, which display 184 and 248 alleles, respectively (Maccari et al. 2020). The full functional implications of this allelic variation are still unknown. For some KIRs, the allele has been shown to impact surface protein levels. For example, *KIR3DL1* alleles have been grouped into “high expressers” and “low expressers” whilst the KIR3DL1*004 molecule is not expressed on the cell surface and is considered a null allele (Boudreau et al. 2014; Gardiner et al. 2001; Pando et al. 2003; Yawata et al. 2006). The binding strength of KIRs to their ligands is also determined by allelic polymorphism; for example, KIRs encoded by the *KIR2DL2* alleles are typically stronger binders than those encoded by the *KIR2DL3* alleles (Moesta et al. 2008). Although KIR A haplotypes are invariant in terms of gene content, they have retained high levels of polymorphism (Pyo et al. 2010). The clinical relevance of KIR allelic variation is demonstrated by disease associations such as the association between possession of the KIR3DL1*004 allele and slower progression to AIDS (Martin et al. 2007) or KIR3DL2*107 and early onset of allergic pathologies (Gao et al. 2022).

Finally, each KIR binds a subset of highly polymorphic HLA class I molecules. Although not all KIR ligands have been identified, especially those for aKIRs, it is well established that iKIRs bind HLA molecules in broad allele groups. For example, KIR2DL1 recognizes HLA molecules carrying the C2 motif (Asn at position 77 and Lys at position 80), while KIR2DL2/L3 molecules recognize C1 group HLA alleles (possessing Ser at position 77 and Asn at position 80) (Biassoni et al. 1997; Valés-Gómez et al. 1998) and KIR3DL1 binds HLA molecules carrying the Bw4 epitope, a sequence motif determined by amino acids 77–83 on the α-1 α-helix (Gumperz et al. 1997). KIRs and their HLA ligands are encoded on different chromosomes and so are inherited independently. This gives rise to a great combinatorial diversity: individuals vary in the number of KIR-HLA pairs they carry as the genes encoding either the ligand, the receptor or both might be missing. Therefore, in addition to KIR and HLA allelic variation, functional KIR polymorphism is ultimately amplified by the combinations inherited from these two unlinked loci.

The KIR genes are rapidly evolving, and whilst present in primates (and a separate lineage in cattle), they are absent in mice (as well as other mammals such as rats and horses). The murine functional homologue of the KIRs is the Ly49 lectin-like receptors (Guethlein et al. 2015). KIR and Ly49 receptors are structurally unrelated (immunoglobulin-like vs C-type lectin-like glycoproteins, respectively), and there are important differences in tissue distribution; for example, Ly49 receptors are expressed on neuronal soma, axons and dendrites where they play a role in neurite branching, synapse formation and neuronal survival (Zohar et al. 2008) but no comparable role of KIR has been described. However, KIR and Ly49 do share some important similarities: they both bind MHC molecules and are both widely expressed in NK cells.

## KIRs and NK cell-mediated immunity

The main role of KIRs is to modulate NK cell-mediated immunity. The importance of this role is highlighted by immunogenetics studies in which carriage of KIR genes together with the genes encoding their ligands has been statistically associated with control of viral infection, increased survival following hematopoietic cell transplant in leukaemia patients, risk of autoimmunity and probability of reproductive success (Hiby et al. 2004, 2014; Jiang et al. 2013; Ruggeri et al. 1999; Traherne et al. 2016). These associations have been recently reviewed in (Pollock et al. 2022) so we only touch on them briefly here. The most well-studied associations between KIR-ligand and clinical outcome are in human immunodeficiency virus 1 (HIV-1) infection. The aKIR allele at the *KIR3DL1/S1* locus, *KIR3DS1*, in the presence of its Bw4-80I ligand, is associated with low setpoint HIV-1 viral load, slower progression to AIDS and low CD4+ T cell count and reduced occurrence of opportunistic infections in different HIV-1 infected cohorts (Barbour et al. 2007; Boelen et al. 2018; Martin et al. 2002; Pelak et al. 2011; Qi et al. 2006). Striking associations have also been reported between *KIR3DS1-Bw480I* and seronegativity in HIV-exposed individuals (Boulet et al. 2008; Jennes et al. 2013). Other examples of KIRs being associated with changes in the course of chronic viral infection include associations with spontaneous clearance of hepatitis C virus infection (Jennes et al. 2013; Khakoo et al. 2004). In the presence of their ligands, KIRs have also been associated with the risk of autoimmune diseases; for example, the *KIR2DL2-C2* compound genotype increases psoriasis risk whereas *KIR3DL1-Bw4* is protective against multiple sclerosis (Ahn et al. 2021; Hollenbach et al. 2016). Finally, certain KIRs have been strongly associated with reproductive complications, including pre-eclampsia and the risk of miscarriage (Hiby et al. 2004; Huhn et al. 2018).

## KIRs and T cell-mediated immunity

In addition to their clear role in modulating NK cell-mediated immunity, there are multiple, non-exclusive pathways by which KIRs could, potentially, modulate T cell-mediated immunity. We divide these pathways into “direct” in which KIR expression on a T cell directly impacts that T cell’s function and survival, and “indirect” in which KIR expression on another cell (e.g. an NK cell or a different T cell) indirectly affects T cell function.

### Direct pathways

When NK cells were shown to kill tumour cells in a peptide-independent MHC-dependent fashion, the field hypothesised the existence of NK receptors responsible for executing the so-called missing self-response (Ljunggren and Kärre 1990). In parallel, a 58 kDa surface receptor, named p58 (KIR2DL2/S2, KIR2DL3), was identified as an inhibitor of NK cell activation (Moretta et al. 1990). Shortly after, the identification of similar inhibitory receptors and their ability to interact with HLA class I molecules changed the view of NK cells (Ciccone et al. 1992; Moretta et al. 1993). From being considered a non-specific effector population, the discovery of the molecular mechanism of the missing-self response confirmed the hypothesis that NK cell specificity was dependent on interactions between HLA molecules and inhibitory NK receptors, the KIRs. Although KIRs are predominantly expressed in NK cells, flow cytometry data that led to the discovery of p58 on NK cells already showed the existence of a minor subset of CD3+KIR+ T cells (Moretta et al. 1990).

Follow-up studies have consistently reported the presence of KIR+ cells in different T cell subsets across healthy individuals. These KIR+ T cell populations are found both within the gamma/delta (Battistini et al. 1997; Nakajima et al. 1995) and the alpha/beta T cell compartments (Ferrini et al. 1994; Mingari et al. 1996; Phillips et al. 1995). Amongst conventional alpha/beta T cells, the highest frequencies of KIR expression are found in CD8+ T cells (1–25%); nevertheless, low frequencies of KIR+CD4+ T cells (< 1%) are also consistently detected (Moretta et al. 1990; Phillips et al. 1995; van Bergen et al. 2004). KIR+CD8+ T cells express memory markers and accumulate in the terminally differentiated compartment with age; up to 25% of CD8+ T cells express KIRs in elderly individuals, an observation that has been replicated for Ly49 receptors in mice (Anfossi et al. 2001; Coles et al. 2000). Interestingly, Ly49+ T cells expand in some disease settings like influenza, lymphocytic choriomeningitis and *Listeria monocytogenes* murine viral infections (Kambayashi et al. 2000; McMahon and Raulet 2001). In humans, KIR + T cells are enriched in HIV and HCV-infected individuals (Bonorino et al. 2007; Cauda et al. 1994), although in HCV infection, KIR+ T cell frequency does not correlate with lesion severity (Bonorino et al. 2007). In HIV-1 infection, KIR expression on CD8+ T cells correlated with RNA viral load and increased with the duration of HIV-1 infection. The subset of individuals with good control of HIV-1 (long-term non-progressors) also had a lower frequency of KIR+CD8+ T cells. Whether the increase in KIR+ cell frequency is a cause or consequence of high viral load cannot be conclusively established in these observational studies but the decrease in KIR+CD8+ T cell frequency amongst individuals on viral suppressive anti-retroviral treatment suggests that it is perhaps viral load that drives KIR expression rather than the converse (Alter et al. 2009). In a recent study of individuals living with chronic HIV-1 infection, KIR+CD8+ T cells negatively correlate with the total HIV-1 reservoir size (DNA load) (Jin et al. 2020). KIR+CD8+ T cell expansion was also detected in haploidentical bone marrow transplant recipients but not in fully compatible transplants (Albi et al. 1996).

The effects of KIR expression on that T cell’s function are generally thought to depend on the presence of KIR ligands. Two main effects of ligation of iKIR expressed by T cells have been reported: inhibition of T cell effector function and reduction of activation-induced cell death (AICD). As might be expected by the nature of the T cell recognition via TCRs, KIR+ T cells do not display a TCR-independent, NK-like activity and are unable to kill cells that lack HLA expression (Guerra et al. 2000). Many studies have shown that KIR engagement with their HLA ligands dampens the TCR-dependent effector response, both via reduction of cytokine levels (IFNg and TNFa) (D’Andrea et al. 1996) and impaired cytotoxicity (Bakker et al. 1998). However, the extent and nature of this inhibition are highly heterogeneous across studies (Anfossi et al. 2004; Ferrini et al. 1994). While some studies argue that KIR modulation of CD8+ T cells is ligand-independent (it has been suggested that this could be explained by a process of education for CD8+ T cells as there is for NK cells) (Alter et al. 2008; Björkström et al. 2012; Chwae et al. 2002), others show a ligand-dependent effect i.e. functional impairment in the presence of cognate HLA (Anfossi et al. 2004; Gati et al. 2003; Guerra et al. 2000, 2002; van der Veken et al. 2009; Zajac et al. 1999). The disparity in these results might be explained by the cell type used (ex vivo KIR+ T cells, KIR+ T cell clones, transgenic KIR+ T cell lines) but also due to the variability of functional KIR receptor repertoire across individuals, the effector function assessed (cytokines vs cytotoxicity) and the nature and concentration of the stimuli. In conclusion, it seems that KIR expression probably reduces activation-dependent T cell effector function, but it is unlikely to be a global inhibitor.

**Fig. 1:**
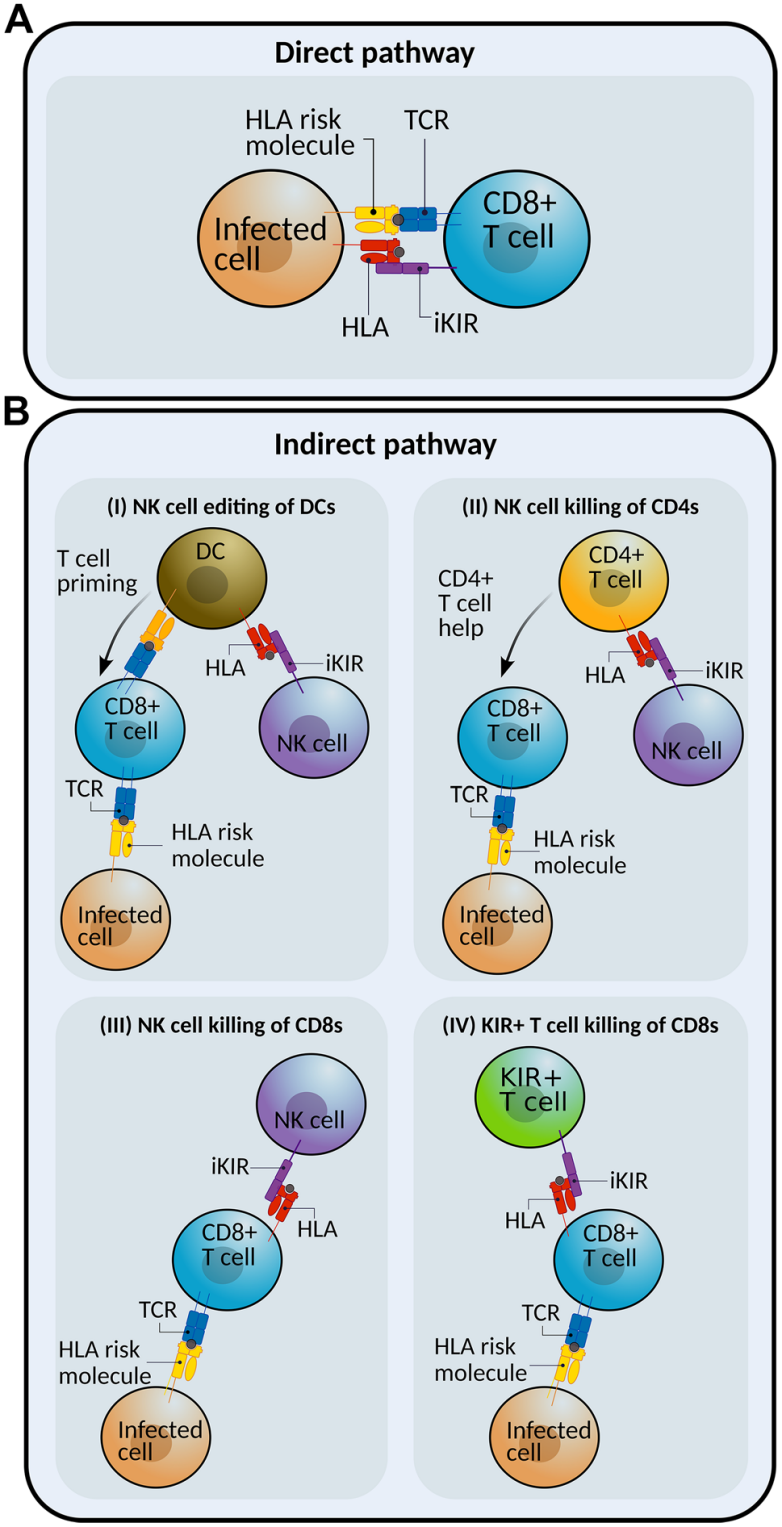
**The direct and indirect pathways**. iKIR could increase T cell survival and lead to an enhancement of HLA class associations by a number of different pathways. In all diagrams, the HLA class I molecule associated with disease outcome is the molecule shown in yellow (interacting with the TCR in blue). **A** Direct pathway: iKIR expression on antigen-specific CD8 + T cells reduces AICD and increases T cell lifespan upon ligation of the cognate KIR ligand. **B** Indirect pathway: iKIR ligation on other cells can affect CD8 + T cell lifespan through different mechanisms. (**I)** NK cells can interact with dendritic cells (DCs) and shape downstream T-cell responses. (**II)** NK cells can directly kill activated CD4+ T cells. (**III)** Similarly, activated CD8+ T cells are also susceptible to NK cell killing. (**IV)** KIR+CD8+ T cells can kill activated antigen-specific CD8+ T cells. iKIR ligation might impair the CD8+ regulatory function, resulting in increased T cell lifespan during autoreactive or antiviral responses

As opposed to the inhibitory KIR effect on T cell function, the reduction of AICD upon KIR ligation is well established (Fig. 1A). Using mice transgenic for KIR2DL3, Ugolini et al. showed that if the mice were also transgenic for the matching HLA ligand, then there was a significant accumulation of memory KIR+CD8+ T cells but this was abrogated in mice that were negative either for the ligand, the KIR or both (Ugolini et al. 2001). Similarly, the size of this memory population increased with age in these transgenic mice, an observation already reported for Ly49+ CD8+ T cells in non-transgenic mice (Coles et al. 2000) and recapitulated in healthy humans (Anfossi et al. 2001). Although KIR ligation had no impact on the number of cell divisions, KIR engagement resulted in reduced AICD in T cells in vitro, suggesting that the accumulation of memory CD8 T cells in vivo was attributable to increased T cell survival (Anfossi et al. 2001; Ugolini et al. 2001). Similar in vitro experiments using the Ly49 receptor in mice resulted in the inhibition of TCR-induced apoptosis in the presence of the Ly49 cognate ligand (Roger et al. 2001). Although in vitro experiments using a transfected KIR3DL1 Jurkat cell line suggested ligation was dispensable (Chwae et al. 2002), in human T cell clones, KIR ligation reduces AICD (Arlettaz et al. 2004). Consistent with this, KIR expression correlates with higher levels of the antiapoptotic molecule Bcl-2 (Young et al. 2001) and decreased caspase 8 activity (Gati et al. 2003).

Although their discovery dates back over 3 decades ago, the functional relevance of the KIR+ T cell population is unclear. Several reasons explain the paucity of functional studies. First, the absence of the KIR family in murine species hinders the translation of in vivo studies. Second, due to the relatively wide expression of KIR, it is difficult to identify which population is responsible for genetic associations. Finally, the antigen specificity of KIR+ T cells is often unknown (Young et al. 2001). Cases where KIR+ T cell specificity has been identified include self-antigen specific T cell clones isolated from renal cell carcinoma (Guerra et al. 2000) and melanoma (Speiser et al. 1999) patients as well as HLA multimer+KIR+ T cells specific for cytomegalovirus, human T cell leukaemia virus (HTLV-1) and human immunodeficiency virus (HIV-1) (Alter et al. 2008; Boelen et al. 2018; van der Veken et al. 2009; Young et al. 2001). Given the specificity of these KIR+ T cell populations and their accumulation in the terminally differentiated compartment, it has been suggested that KIRs on T cells might be involved in the regulation of autoreactive responses (Alter et al. 2008; Cauda et al. 1994; Guerra et al. 2000), acting as an immune checkpoint that increases the activation threshold of potentially autoreactive T cells. KIR+ T cells can also play an indirect role by regulating other T cell populations. KIR+ T cell regulatory function is covered in the next section on indirect pathways.

### Indirect pathways

A set of alternative pathways whereby iKIRs indirectly affect CD8+ T cell function and survival is through iKIR expression on other immune effectors (e.g. NK cells or other T cells) interacting with the responding T cell population (Fig. 1B).

There are many ways in which NK cells shape adaptive immunity. For example, NK cells interact with dendritic cells (DCs) during the T cell priming phase, a process known as NK-DC crosstalk (Fig. 1B(I)). There is evidence that NK-DC crosstalk can have both a positive effect (boosting the subsequent T cell response) or a negative effect, dampening it down. Upon activation, NK cells secrete cytokines including interferon-gamma, which enhance dendritic cell maturation (Piccioli et al. 2002). Furthermore, during viral infection, NK cells have been shown to kill DCs, so-called DC editing, which ensures robust T cell responses by selectively depleting immature DCs and sparing the most immunogenic DCs (Ferlazzo et al. 2002; Morandi et al. 2012; Piccioli et al. 2002). It has also been shown that NK-DC crosstalk during priming can have the opposite effect and lead to impairment of the T cell response since NK killing of immature DCs can lead to a reduction in the efficacy of DC vaccinations (Hayakawa et al. 2004) and impairment of viral control (Andrews et al. 2010; Cook and Whitmire 2013; Mandaric et al. 2012). Whether NK-DC crosstalk promotes or dampens effective T cell responses remains unknown, but several factors such as cytokine milieu or the density of co-stimulatory molecules might be important. For example, some studies have shown that KIRs at least partly modulate DC-NK interactions (Chiesa et al. 2003; Ferlazzo et al. 2002), so variability in the KIR-HLA interactions might partly explain the different outcomes.

NK cells can also affect adaptive immunity by killing activated T cells in a perforin-dependent manner. Although activated CD8+ and CD4+ , including Tregs, upregulate activating ligands for NK cells and/or death receptors for NK cells (Cerboni et al. 2007; Crouse et al. 2014; Nielsen et al. 2012; Peppa et al. 2013; Rabinovich et al. 2003; Soderquest et al. 2011; Waggoner et al. 2012; Welsh and Waggoner 2013), the exact mechanisms of NK recognition and killing of T cells are still not well defined (Waggoner et al. 2016). It has been suggested that the selectivity for activated cells indicates that NK cells might kill incorrectly activated T cells such as autoreactive T cells (Nielsen et al. 2012). Indeed, NK cells control autoreactive CD4+ T cells and ameliorate inflammation in a mouse model of multiple sclerosis (Laroni et al. 2016; Lu et al. 2007). In murine LCMV infection, NK cell killing of CD4+ T cells has been shown to be protective and to prevent immunopathology in the context of high viral load (Waggoner et al. 2012) (Fig. 1B(II)). However, in the same study, when using an intermediate viral dose, NK cell regulation results in viral persistence and immune pathology (Lang et al. 2012; Soderquest et al. 2011; Waggoner et al. 2012). Therefore, while NK cell dampening of CD4+ T cell responses might be beneficial in some contexts (autoimmunity, immunopathology), it can also have long-term negative effects (including viral persistence) and result in a reduced T cell memory pool (Lu et al. 2007; Rydyznski et al. 2015; Soderquest et al. 2011). NK killing of CD8+ T cells has also been described by Peppa et al. (Fig. 1B(III)). Using PBMCs from chronic hepatitis B virus patients (HBV), they showed that, in vitro, NK cells selectively kill HBV-specific T cells via the engagement of death receptors (Peppa et al. 2013). This regulatory role of NK cells on CD8+ T cells has been recently shown to limit specific CD8+ T cell responses to HBV vaccine in mouse in vivo and in humans in vitro in a programmed death ligand 1-dependent manner (Diniz et al. 2022).

The role of iKIRs during NK cell killing of activated T cells has not been addressed. NK-T cell interactions probably take place within secondary lymphoid organs; after T cell priming, T cells undergo clonal expansion for approximately 2 days, coinciding with the peak of NK cell activation. Although perforin+ NK cells do not reside in secondary lymphoid organs (Dogra et al. 2020), upon activation during viral infections, type I interferon signalling triggers NK cell accumulation and renders them in close contact with T cells (Ali et al. 2021). Interestingly, NK cells within lymph nodes not only acquire cytolytic functions but also KIR expression (Ferlazzo et al. 2004). Therefore, activated KIR + NK cells are probably interacting with recently activated T cells in the lymph nodes, and the outcome of this interaction might be modulated by the iKIR-HLA receptor-ligand system. Type I interferon also induces KIR ligand expression, HLA-I, in activated T cells so T cells might avoid NK cell killing via iKIR engagement (Xu et al. 2014).

NK cells are not the only KIR-expressing effector population that can affect T cell survival (Fig. 1B(IV)). A recent study from Li et al. reports that KIR-expressing T cells can also regulate other T cell populations (Li et al. 2022). They observed that KIR+CD8+ T cells are expanded in different autoimmune conditions and also in patients with severe COVID-19. A previous study also observed that activated KIR+CD45RA+ T cells expand during acute respiratory infection (including COVID-19 patients) and showed that KIR+RA+ T cells suppress the proliferation of stimulated KIR- CD8 T cells in vitro (Pieren et al. 2021). To address the function of the KIR+CD8+ T cells in autoimmunity, Li et al. isolated gliadin-reactive CD4+ T cells from celiac patients and cocultured them with KIR+CD8+ T cells. Upon activation, KIR+CD8+ T cells killed activated CD4+ T cells in a perforin-dependent manner. This effect was partly abrogated by the presence of anti-HLA class I antibodies and non-classical anti-HLA-E antibodies. Whether the interaction between HLA molecules and the relevant KIR is modulating this regulatory function was not addressed, and although the KIR+ T cells were preactivated before blocking, classical HLA class I blockade could in principle be attributed to TCR signalling, KIR signalling or both. Mice lacking Ly49+CD8+ T cells displayed normal antiviral responses but were more prone to autoimmunity. These observations are built on previous work on mouse models of multiple sclerosis where Ly49+ T cells were shown to suppress disease (Jiang et al. 1992; Saligrama et al. 2019). Based on this work, it was argued that KIR+CD8+ T cells are the counterpart of CD4+ regulatory T cells (Tregs) and might limit autoreactivity and immunopathology during infection.

## Clinical relevance

Whilst there are multiple pathways whereby KIRs could affect T cell responses, whether these interactions have any impact on the course of disease in humans has not been studied. We have recently conducted an extensive immunogenetic analysis of three different persistent viral infections to address this question (Boelen et al. 2018; Seich al Basatena et al. 2011). In contrast to KIR-HLA association studies (which address the impact of KIR modulation of NK cells on the course of the disease), we start with the subset of classical HLA class I disease associations which are attributable to T cells; these associations indicate that CD8+ T cells have a significant impact on clinical outcome. If iKIR-HLA ligand interactions modulate CD8+ T cell responses, then we would predict that functional iKIR genes (iKIR genes together with their HLA ligand genes) would modify these T cell-mediated HLA class I disease associations.

The interaction we are interested in investigating is therefore a three-way (gene–gene-gene) interaction, namely the iKIR gene—iKIR ligand gene—disease risk HLA allele. Three-way gene interactions are not investigated in GWAS studies because the resulting explosion of multiple comparisons is prohibitive; the interaction of interest here is thus invisible to hypothesis-free GWAS approaches and would have been missed by GWAS studies to date (Benjamini and Hochberg 1995). To detect such three-way gene interactions, hypothesis-driven i.e. candidate gene studies in moderate to large cohorts are required. To investigate the clinical relevance of iKIR- T cell interaction, we analyzed three independent cohorts of individuals living with persistent viral infections: hepatitis C virus (HCV), HIV-1 and HTLV-1 (for details on cohort sizes, ethnic origin and outcome metrics analysed, see Table S1). In the following sections, we review the immunogenetics of these persistent viral infections starting with well-known HLA class I associations followed with a review of findings from others and ourselves that these associations are modified by KIR-HLA ligand pairs i.e. there is a three-way association between iKIR genes, ligand genes and HLA class I risk alleles. This is consistent with the hypothesis that iKIR interactions with their ligands modulate HLA-restricted CD8 + T cell responses during these three chronic viral infections.

### Classical HLA class I disease associations in chronic viral infections

The HLA genes are located within the major histocompatibility complex (MHC) region in chromosome 6 and are a known hotspot for disease associations. Some of the most well-documented associations are between certain HLA class I alleles and clinical outcomes of chronic viral infections such as HIV-1 and HCV. For example, HLA-B*57 is associated with low setpoint viral load and slow progression to disease in multiple HIV-1 cohorts (Carrington et al. 1999; Kaslow et al. 1996; Martin et al. 2002). In HCV, HLA-B*57 is also protective, and it is associated with spontaneous clearance of infection (Chuang et al. 2007; Thio et al. 2002). Detrimental HLA allele associations have also been reported; in HIV-1 infection, a subset of HLA-B*35 alleles, HLA-B*35Px (Gao et al. 2001), is associated with high setpoint viral load and faster progression to disease (Carrington et al. 1999). In individuals living with HTLV-1 infection, HLA-A*02:07, HLA-A*02:06 and HLA-C*08 are associated with low proviral load and reduced risk of the inflammatory disease HTLV-1-associated myelopathy/tropical spastic paraparesis (HAM/TSP) whereas HLA-B*54:01 is detrimental i.e. it is associated with a significantly higher proviral load and an increased risk of HAM/TSP (Jeffery et al. 1999). Other HLA associations have been reported for these chronic viral infections but have not been consistently reproduced in different populations highlighting the importance of controlling for confounding, especially linkage disequilibrium and population stratification (Carrington and Alter 2012).

HLA class I molecules bind a range of molecules: TCR, iKIR, some aKIR as well as the leucocyte immunoglobulin-like receptors LILRB1 and LILRB2. Because of this pleiotropic role, the mechanistic interpretation of HLA class I disease associations is not straightforward. Since we wished to investigate if KIRs affected HLA associations as a proxy for KIRs affecting T cell responses, we focused on the subset of HLA class I associations that were attributable to T cells. Initially, we identified HLA associations which could be attributed to T cells in an ad hoc way (Boelen et al. 2018; Seich al Basatena et al. 2011). For example, the detrimental association between HLA-B*54:01 and HTLV-1 proviral load is unlikely to be explained by the B54:01 molecule’s status as a KIR ligand because HLA-B54 is not known to bind any KIR and other HLA alleles with the same motif in the KIR binding region (the so-called Bw6 alleles) are not detrimental in the context of HTLV-1 infection. Similarly, other HLA molecules with similar LILR binding to B*54:01 are also not detrimental. More recently, we performed this analysis in a more systematic way by developing an algorithm, *fstool* (Debebe and Asquith 2020), to identify and quantify the relative contribution of different receptor-ligand interactions to HLA class I disease associations (Debebe et al. 2020). Briefly, Debebe et. al. developed metrics quantifying the similarity of HLA class I alleles to each other in terms of their TCR binding, activating and inhibitory KIR binding and LILRB1 and LILRB2 binding. Then, they used multiple regression to quantify the association between similar HLA class I alleles and clinical outcomes. They hypothesized that if the underlying mechanism of a given HLA class I association is attributable to the CD8+ T cell response, other HLA alleles with similar TCR binding would show a similar disease association, and consequently, the TCR similarly metric would be significantly associated with outcome but HLA alleles with similar iKIR binding (for instance) would not be significantly associated with the disease. The same reasoning follows for the other mechanisms i.e. KIR and LILR-mediated responses. For HTLV-1 infection, the results were striking: all 4 HLA class I disease associations tested were best explained by TCR binding. Indeed, it was not just the behaviour of the 4 extreme protective or detrimental alleles that was explained by TCR binding, the protection conferred by the vast majority of all HLA class I alleles was also clearly attributable to TCR binding. Consistent with this, protective HTLV-1 alleles have been shown to preferentially present epitopes from the HBZ protein (MacNamara et al. 2010). In HCV infection, the picture was less stark with the protection conferred by different alleles explained by different mechanisms. Of note, the protective association with HLA-B*57 was attributable to its TCR-binding properties. Similarly, in HIV-1 infection, HLA-associated protection was mediated by different mechanisms. The protective effect of HLA-B*57 in HIV-1 was mainly attributed to binding to the activating KIR, KIR3DS1, consistent with previous interpretations (Martin et al. 2007). However, once individuals with *KIR3DS1* were excluded from the cohort, there was still a residual protective effect of HLA-B*57, and this was attributable to TCR binding (Debebe et al. 2020). In short, it was possible to identify a number of HLA class I disease associations in HIV-1, HCV and HTLV-1 infections that were most likely attributable to CD8+ T cell responses.

### Functional iKIR genes enhance HLA associations

To investigate the clinical relevance of an interaction between iKIRs and T cells in vivo, Seich al Basatena et al. (2011) performed an immunogenetic analysis of HCV and HTLV-1 cohorts. The study focused on the subset of HLA class I associations which could clearly be attributed to the CD8+ T cell response: HLA-B*57 in HCV and HLA-A*02:07, HLA-A*02:06, HLA-B*54:01 and C*08 in HTLV-1. To test for an interaction between iKIRs and HLA class I restricted T cell responses, the cohorts were stratified into iKIR gene carriers and non-carriers, and the strength of the HLA associations with clinical outcome (namely risk of HAM/TSP in the HTLV-1 cohort and odds of spontaneous clearance in the HCV cohort) was assessed in each stratum. The iKIR gene *KIR2DL2* was found to enhance both protective and detrimental HLA class I associations with clinical outcomes. Additionally, *KIR2DL2* presence also enhanced the protective effect of binding HBZ peptides in HTLV-1 and HLA class I associations with viral load in both HCV and HTLV-1 cohorts. This enhancement was stronger in the presence of genes encoding the stronger KIR2DL2 ligand, (HLA alleles carrying the C1 motif), suggesting that iKIR ligation is important for the observed KIR2DL2 impact on the T cell response. Together, these observations showed for the first time that the iKIR genotype has a significant impact on HLA class I associations suggesting that iKIRs affect antiviral HLA-restricted T cell responses in humans in vivo. No effects were detected for similar iKIRs genes like *KIR2DL1* or *KIR2DL3.* However, the power to detect a significant interaction between these iKIR genes and HLA class I alleles was low compared to that for the *KIR2DL2* gene due to the unbalanced frequency of functional *KIR2DL1* and *KIR2DL3*.

Another potential example of iKIR modulation of HLA risk alleles can be found in a study investigating the role of KIR3DS1 in HIV-1 control (Pelak et al. 2011). Although the expectation was that both *KIR3DL1* and *KIR3DS1* genes, in the presence of their ligand (Bw4 alleles and Bw480I alleles, respectively), would be protective as reported in two previous studies (Martin et al. 2002, 2007), in this cohort only the *KIR3DS1-Bw480I* compound genotype was independently associated with lower viral load. Interestingly, while the *KIR3DL1-Bw4* compound genotype was not protective in this cohort, it was strongly associated with low viral load in a subset of *KIR3DS1*+*Bw480I*+ carriers in a dose-dependent manner. HLA-B*57 carries the Bw480I motif and is enriched among *KIR3DS1*+*Bw480I*+ individuals. Pelak et. al. attributed these findings to an epistatic interaction between *KIR3DL1* and *KIR3DS1* alleles on NK cells, with consequences for innate antiviral responses. An alternative explanation is that KIR3DL1 in the presence of ligand, Bw4, enhances the well-known protective association of HLA-B*57 (a Bw4-80I allele) with low viral load (Gao et al. 2010; Kiepiela et al. 2004). The potential *KIR3DL1* gene enhancement of B*57 protection in this study is reminiscent of the effect we observed in HCV and HTLV-1 with the *KIR2DL2* gene (Seich al Basatena et al. 2011), suggesting a universal mechanism of iKIR modulation of T cell responses.

**Fig. 2:**
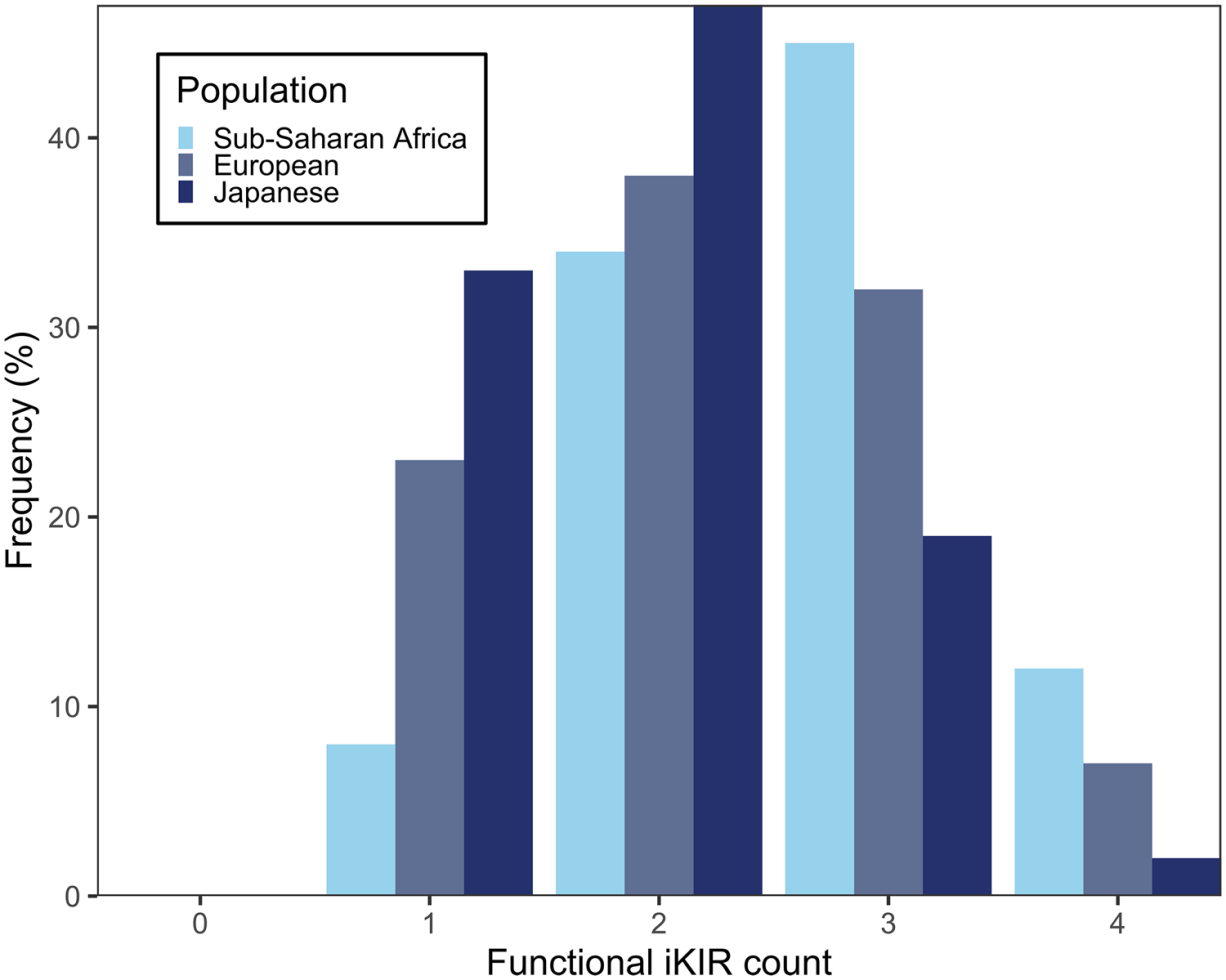
**Functional iKIR gene count distribution differs by ethnicity**. Functional iKIR gene count was calculated from imputed KIR-HLA genotypes from sub-Saharan, European and Japanese individuals. Only *KIR2DL1*, *KIR2DL2*, *KIR2DL3* and *KIR3DL1* genes were considered for iKIR gene count calculation as explained in the text (see the “Functional iKIR genes enhance HLA associations” section). The sub-Saharan and Japanese cohorts were genotyped previously (Jeffery et al. 1999; Martin et al. 2002; Prentice et al. 2014; Seich al Basatena et al. 2011). KIR and HLA genotypes from European individuals were imputed with HLA*IMP and KIR*IMP programs using HumanHap300 and HumanHap610Q SNP data (Motyer et al. 2016; Vukcevic et al. 2015)

To test this hypothesis, in a follow-up study, we extended the study of the KIR2DL2 effect to other iKIRs. There are 6 iKIR loci in the human genome: *KIR2DL1*, *KIR2DL2/3*, *KIR2DL4*, *KIR2DL5*, *KIR3DL1*, *KIR3DL2* and *KIR3DL3*. As mentioned previously, despite having a long cytoplasmatic tail, *KIR2DL4* transduces activating signals (Faure and Long 2002; Kikuchi-Maki et al. 2005), and so this KIR was excluded. The ligands for *KIR2DL5* and *KIR3DL3* have only been discovered recently so were not studied at the time of the analysis (Bhatt et al. 2021; Husain et al. 2019). Additionally, these ligands are not HLA class I molecules so whether the ligation of KIR2DL5 and KIR3DL3 triggers a similar effect compared to the rest of the iKIRs requires further study. And finally, *KIR3DL2*, a framework gene, was excluded since there is considerable evidence that it behaves differently to the other iKIRs (Ridley et al. 2016). We, therefore, focused our analysis on *KIR2DL1*, the two alleles at the *KIR2DL2/3* locus and *KIR3DL1* allele at the *KIR3DL1/S1* locus. Given the functional evidence that iKIR signalling depends on ligation, we defined *functional iKIR genes* as the presence of a given iKIR gene together with the gene encoding the corresponding ligand in the same individual. We then calculated the presence or absence of functional *KIR2DL1*, *KIR2DL2*, *KIR2DL3* and *KIR3DL1* genes. This allowed us to count the number of functional iKIR genes carried by each individual. The number of functional iKIR genes in an individual can take values between zero and four, and its distribution in the population varies with ethnicity (Fig. 2). Related to this count, we also constructed an inhibitory score (iKIR score), a weighted version of the count, that reflects subtleties in each iKIR ligation strength; for example, *KIR2DL2* binding of C1 alleles is stronger on average than *KIR2DL3*. Of note, neither iKIR count nor iKIR score was significantly associated with outcome, ruling out an innate NK cell mediated effect. As expected, the functional KIR gene *KIR3DS1* was associated with low early viral load set point in HIV infected individuals. Carriers of the functional *KIR3DS1* gene were therefore excluded from the analysis to avoid confounding by NK cell-mediated associations.

We then split the cohorts based on the individuals’ inhibitory scores into individuals with a high inhibitory score and individuals with a low inhibitory score, and the strength and significance of HLA associations were assessed in each subcohort (Table S2). All HLA class I associations in this HTLV-1 cohort were strengthened in individuals carrying a high inhibitory score and were greatly weakened and nonsignificant in individuals with a low inhibitory score. Similar results were found when stratifying the cohort based on the count of functional iKIR genes instead. In HCV infection, a similar result was found: the protective HLA-B*57 association was enhanced in individuals with a high iKIR score. Finally, in HIV-1 infection, both the protective HLA-B*57 and detrimental HLA-B*35Px associations were enhanced in individuals with a high iKIR score; this was replicated in two independent cohorts (Boelen et al. 2018). Together, this extensive immunogenetic analysis of three different chronic viral infections validated our earlier preliminary findings that iKIRs, in the presence of their ligands, modulate HLA class I associations, and our interpretation is that this is explained by iKIR modulation of HLA class I restricted T cell responses.

We proposed that iKIRs enhancement of CD8 + T cell survival is a plausible mechanism that can explain this iKIR effect on HLA class I associations. This hypothesis stems from ours and others’ experimental studies on T cells, where iKIR ligation is associated with increased T cell survival either via a direct or indirect pathway (see “Direct pathways” and “Indirect pathways” sections) (Boelen et al. 2018; Ugolini et al. 2001). Although it is difficult to imagine how increased survival can enhance both protective (HLA-B*57) and detrimental (HLA-B*35Px) associations, using mathematical modelling we showed that if T cell lifespan is very short (low number of functional iKIRs), then the effect of protective, detrimental and average HLA class I alleles is indistinguishable; individuals have similar viral loads irrespective of their HLA class I genotype. That is, if we stratified a cohort and looked just at people with a low number of functional iKIRs, then the viral load would be independent of HLA genotype and HLA disease associations would be weak or absent. However, if T cell lifespan increases i.e. in individuals carrying a high number of iKIRs, the quality of the T cell response now becomes much more important and significant differences in viral load emerge between carriers of protective, detrimental and average HLA alleles. In an immunogenetic analysis, this is observed as strong detrimental and protective HLA associations. That is the strength of both protective and detrimental associations would be higher in people with a high iKIR score than in people with a low iKIR score. Although the model does not prove the hypothesis, it does provide a plausible explanation for our seemingly contradictory immunogenetic results.

Although this immunogenetic analysis suggests that iKIRs have a clinically significant impact on T cell responses, it does not distinguish between the direct and the indirect pathways: both are consistent with the data. The only hint is given by the size of the clinical effect compared to the size of the KIR+ T cell population: it is perhaps difficult to imagine how the increase in survival of such a small population of KIR+ T cells can underlie such profound clinical effects, and for this reason, the data are arguably more aligned with the indirect pathway.

## Conclusions

Functional iKIR genes enhance HLA class I disease associations in three different chronic viral infections. In contrast to many reported iKIR-disease associations, these observations applied to all iKIR genes and to all the three viral infections studied. This suggests the existence of a clinically significant checkpoint regulator of T cell responses. Our hypothesis is that the iKIR-HLA receptor-ligand system enhances T cell survival. Enhanced T cell lifespan might be desirable in the context of chronic antigen stimulation to avoid exhaustion and cell death but increased T cell survival might also promote autoreactive T cell responses and worsen autoimmunity. Although some T cells express KIRs, and this may directly affect the T cell’s lifespan (Fig. 1A) perhaps a more plausible explanation is modulation by another KIR expressing immune effector (most likely NK cells but also potentially another T cell subpopulation, Fig. 1B). A number of regulatory mechanisms by which innate cytotoxic effectors affect adaptive responses have been described though the role of iKIRs in these interactions has generally not been investigated. The combination of both mathematical models and experimental data will help to investigate the underlying mechanisms behind our immunogenetic findings.

## Supplementary Information

Below is the link to the electronic supplementary material.

Supplementary file1 (DOCX 20 KB)
