# Supplementary material for: Innate receptors modulating adaptive T cell responses: KIR-HLA interactions and T cell-mediated control of chronic viral infections
Source: Immunogenetics. 2023 Jan 31;75(3):269–82. doi: 10.1007/s00251-023-01293-w (PMC9887252; doi:10.1007/s00251-023-01293-w)
Supplement: Supplementary file 1 — Supplementary file1 (DOCX 20 KB) [file 251_2023_1293_MOESM1_ESM.docx]

## Supplementary Information

|  | **Cohort** | | **N** | **Ethnic origin** | **Outcome metric** |
| --- | --- | --- | --- | --- | --- |
| 1 | IAVI (HIV-1 seroconverters) | | 491 | sub-Saharan Africa | Early viral load set point |
|  |  |  | 461 |  | time to low CD4 count |
| 2 | IAVI–partners (HIV-1 seropositives) | | 391 | sub-Saharan Africa | Median log_10_ viral load |
| 3 | CHAVI (HIV) | | 177 | sub-Saharan Africa | Median log_10_ viral load |
| 4 | US (HIV) | | 548 | 57.8% white 38.7% African American  3.5% Hispanic/other | time to CD4+ T cell count<200 cells/mm^3^ |
| 5 | Kagoshima (HTLV-1 seropositives) | | 392  (214 HAM/TSP,  178 asymptomatic carriers) | Japanese | HAM/TSP patient |
| 6 | HCV (HCV seropositives) | |  | Caucasian | Spontaneous clearance of HCV |
|  |  | AIDS Link to Intravenous Experience (ALIVE) | 226 |  |  |
|  |  | Multicenter Hemophilia Cohort Study (MHCS) | 295 |  |  |
|  |  | Hemophilia Growth and Development Study (HGDS) | 100 |  |  |
|  |  | UK cohort | 161 |  |  |

**Table S1. Cohorts analysed in** (Boelen et al., 2018; Seich al Basatena et al., 2011)**.**

| **Cohort** | **Viral infection** | **Outcome metric** | **iKIR score threshold** | **HLA**  **association** | **HLA in iKIR lo** | | **HLA in iKIR hi** | | **N** | |
| --- | --- | --- | --- | --- | --- | --- | --- | --- | --- | --- |
|  |  |  |  |  | **Effect** | **P value** | **Effect** | **P value** | **HLA^+^** | **HLA^-^** |
|  | | | | | | | | | | |
| Pooled IAVI and IAVI partners | HIV-1 | Log viral load | 2 | *B*57* | Coeff=−0.33 | 0.08∙ | Coeff=−0.47 | 0.0002*** | 91 | 750 |
| Pooled IAVI and IAVI partners | HIV-1 | Log viral load | 2 | *B*35Px* | Coeff=−0.12 | 0.4 | Coeff=+0.31 | 0.003** | 153 | 688 |
| US | HIV-1 | Time to low CD4 count (<200 cells/mm^3^) | 2 | *B*57* | HR=0.29 | 0.25 | HR= 0.25 | 0.004** | 44 | 446 |
| US | HIV-1 | Time to low CD4 count (<200 cells/mm^3^) | 2 | *B*35Px* | HR=1.54 | 0.22 | HR=2.57 | 0.001** | 64 | 426 |
| Kagoshima | HTLV-1 | Odds of HAM/TSP | 1.5 | *B*54* | OR=1.29 | 0.7 | OR=5.33 | 0.0007*** | 82 | 310 |
| Kagoshima | HTLV-1 | Odds of HAM/TSP | 1.5 | *A*02:07* | OR=0.81 | 0.8 | OR=0.10 | 0.003** | 30 | 362 |
| Kagoshima | HTLV-1 | Odds of HAM/TSP | 1.5 | *C*08* | OR=1.09 | 0.9 | OR=0.32 | 0.02* | 56 | 336 |
| HCV-seropositives | HCV | Odds of spontaneous clearance of HCV | 2.5 | *B*57* | OR=1.55 | 0.3 | OR=2.04 | 0.03* | 84 | 697 |

**Table S2. iKIR effect on HLA class I associations in chronic viral infections.** The effect of iKIR score (weighted sum of functional iKIR genes) on HLA class I associations was assessed in the cohorts listed in **Table S1**. IAVI and IAVI partners cohorts were pooled to increase cohort size. Each cohort was stratified into individuals with high and low iKIR score according to a set cut-off (chosen to give balanced groups). The risk of the HLA class I alleles significantly associated with clinical outcome was re-assessed in each stratum i.e in individuals with a high iKIR score (denoted **iKIR hi**) and in individuals with a low iKIR score (denoted **iKIR lo**). The measure of association (**Effect**) varies according to the outcome metric: coefficient of regression (**Coeff**) for log viral load, hazard ratio (**HR**) for time to low CD4 count and odds ratio (**OR**) for odds of disease or odds of spontaneous clearance. The effect sizes for each HLA association in each iKIR stratum are reported alongside p-values and cohort sizes. Analysis with groups based on the count of functional iKIR genes rather than iKIR score gave very similar results (the two numbers are highly correlated). Additional analyses considering each iKIR independently, using non-pooled cohorts and with different iKIR score cutoffs are reported in the original studies (Boelen et al., 2018; Seich al Basatena et al., 2011). P < 0.001 ***; P < 0.01 **; P < 0.05 *; P < 0.1 ·; P values are two-tailed.
